# Estimation of Rice Aboveground Biomass by UAV Imagery with Photosynthetic Accumulation Models

**DOI:** 10.34133/plantphenomics.0056

**Published:** 2023-05-31

**Authors:** Kaili Yang, Jiacai Mo, Shanjun Luo, Yi Peng, Shenghui Fang, Xianting Wu, Renshan Zhu, Yuanjin Li, Ningge Yuan, Cong Zhou, Yan Gong

**Affiliations:** ^1^ School of Remote Sensing and Information Engineering, Wuhan University, Wuhan, China.; ^2^ Lab for Remote Sensing of Crop Phenotyping, Wuhan University, Wuhan, China.; ^3^ College of Life Sciences, Wuhan University, Wuhan, China.

## Abstract

The effective and accurate aboveground biomass (AGB) estimation facilitates evaluating crop growth and site-specific crop management. Considering that rice accumulates AGB mainly through green leaf photosynthesis, we proposed the photosynthetic accumulation model (PAM) and its simplified version and compared them for estimating AGB. These methods estimate the AGB of various rice cultivars throughout the growing season by integrating vegetation index (VI) and canopy height based on images acquired by unmanned aerial vehicles (UAV). The results indicated that the correlation of VI and AGB was weak for the whole growing season of rice and the accuracy of the height model was also limited for the whole growing season. In comparison with the NDVI-based rice AGB estimation model in 2019 data (*R*^2^ = 0.03, RMSE = 603.33 g/m^2^) and canopy height (*R*^2^ = 0.79, RMSE = 283.33 g/m^2^), the PAM calculated by NDVI and canopy height could provide a better estimate of AGB of rice (*R*^2^ = 0.95, RMSE = 136.81 g/m^2^). Then, based on the time-series analysis of the accumulative model, a simplified photosynthetic accumulation model (SPAM) was proposed that only needs limited observations to achieve *R*^2^ above 0.8. The PAM and SPAM models built by using 2 years of samples successfully predicted the third year of samples and also demonstrated the robustness and generalization ability of the models. In conclusion, these methods can be easily and efficiently applied to the UAV estimation of rice AGB over the entire growing season, which has great potential to serve for large-scale field management and also for breeding.

## Introduction

Aboveground biomass (AGB) plays an essential contribution to the carbon pool of an ecosystem [1,2] and is one of the most important indicators for estimating crop yield [3,4]. Effective, accurate, and rapid monitoring of rice AGB in localized areas can support decision-making for high-throughput screening of crop breeding materials [5,6] and assist agricultural managers to improve farmland management [3,7,8]. Rice (*Oryza sativa* L.) is one of the paramount necessities of the people, which provides food for over half of the worldwide population [9,10]. The dynamic, accurate, and rapid monitoring of AGB provides an important reference for assessing the growth status and yield of rice.

The direct methods usually obtain AGB by manual sampling and weighing plants, which can cause damage to vegetation, require a lot of labor, and waste time [3,11,12]. The use of remote sensing technology, especially using unmanned aerial vehicles (UAV) to estimate vegetation AGB, which enables timely and nondestructive crop status assessments, has been widely implemented for crop monitoring and management in large areas for long time series [5,13–15].

Healthy vegetation exhibits specific interactions with certain wavelengths in the electromagnetic spectrum [16,17]. In the visible region, chlorophyll mainly absorbs energy at red and blue wavelengths for photosynthesis and reflects energy at green wavelengths. In the near-infrared wavelengths, on the other hand, because of the structural characteristics of vegetation, it exhibits strong reflection. Therefore, this distinctive spectral characteristic of vegetation has motivated many researchers to explore developing vegetation indices (VIs) and quantitative estimation of vegetation using remote sensing images [18–24]. Using spectral VIs calculated by mathematical combinations of reflectance within several bands is an efficient approach to the biomass estimation of rice [25–27], winter wheat [28], and maize [29]. However, the relationship between VI and biomass is nonlinear, with VI tending to saturate when biomass is high [18,30]. In recent years, some new VIs such as EVI2 [31] have proposed to improve this problem by adjusting the combination of different bands, but saturation still exists. Machine learning methods [23] and deep convolutional neural networks [32] could handle complex and nonlinear data well when estimating crop AGBs. Nevertheless, these models are complex and require many parameters [33] and high computational costs [13,34], making them difficult to apply. On the other hand, the most commonly used VIs are potentially relevant to the agronomic traits during the vegetative phase but tend to lose their sensitivity when the panicles emerge [10,35]. The appearance of the panicle causes a remarkable change in the color of the canopy, while the roughness of the panicle further increases the complexity of the canopy. This poses many new challenges for the description of vegetation growth using VI.

Besides spectral information, canopy structure parameters, such as canopy height, are often used to estimate crop AGB [36,37]. In recent years, there has been an increasing amount of literature on using remote sensing means such as 3-dimensional (3D) point clouds derived from RGB images [3,38] or LiDAR [36,39,40] to obtain canopy height and then to estimate crop biomass. However, Li et al. [3] found that model accuracy was cultivar dependent in potato biomass estimation. In recent years, breeding techniques have produced hundreds of rice cultivars with various phenology cycles [41,42]. There is uncertainty about whether the canopy height can still be effective to estimate multi-cultivar rice AGB in breeding.

Photosynthesis is the foundation of biomass generation, and these photosynthetic materials provide the carbon skeleton that assembles the entire plant [43,44]. The dry matter production is primarily determined by photosynthetic efficiency, leaf area index (LAI), and the duration time during which photosynthesis continues [45]. LAI is defined as the area of total leaves in each unit of the land area [46–48] and is also a key parameter to characterize crop growth [49]. Gong et al. [50] analyzed the complementarity of height and VIs in estimating LAI throughout the entire season at the statistical level and established a simple model for estimating rice LAI with high accuracy and universality by combining height with VI estimation models. Together, these studies provide important insights to estimate the rice AGB throughout the whole growth season by using the idea of photosynthesis accumulation.

Considering the inadequacy of existing estimates of rice biomass for the whole growing season using only a single index such as VI and height, a new accumulative model was proposed by integrating VI and canopy height estimated from UAV images to improve the generality and accuracy of biomass estimation for the whole growing season. The main purpose of this study is as follows: (a) constructing a vertical distribution model for rice to accurately estimate LAI by combining height and VIs, (b) developing a photosynthetic accumulation model (PAM) to estimate rice AGB accurately, (c) proposing a simplified photosynthetic accumulation model (SPAM) while ensuring the precision of rice biomass estimation, and (d) testing the transferability of the 2 models using data acquired in different years, comparing the performance of different methods in predicting AGB, and providing suggestions for their application under different needs.

## Materials and Methods

### Field experimental design

**Fig. 1.**
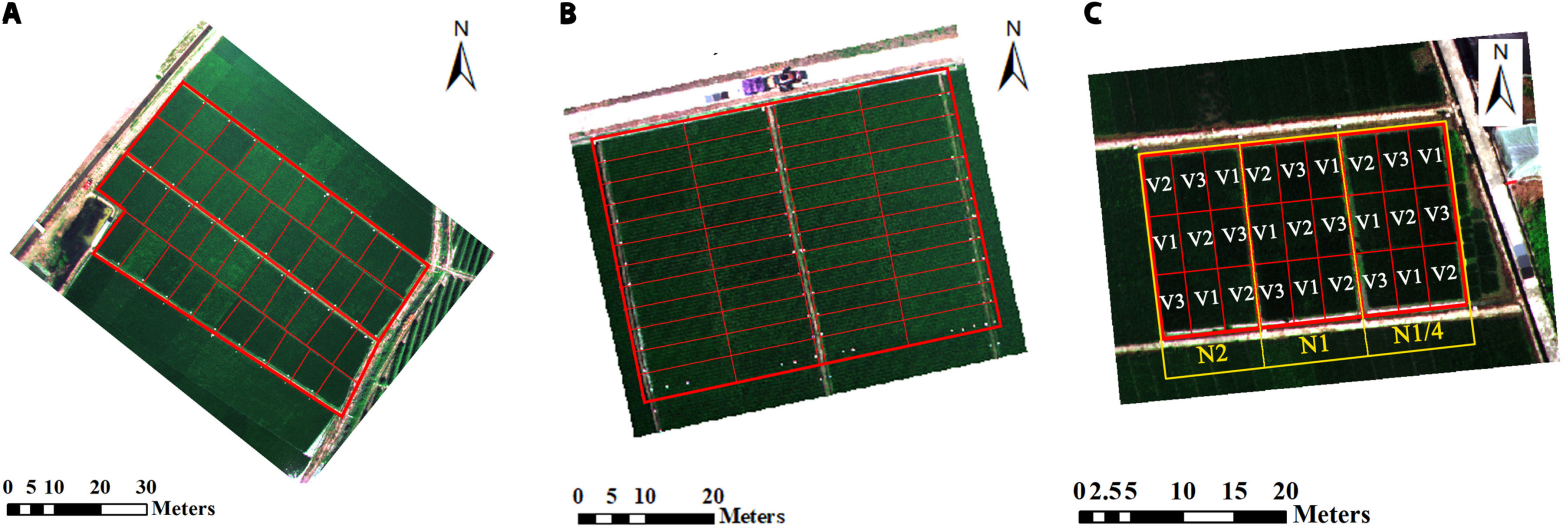
Study area. (A) Experiment 1 was conducted in Lingshui, Hainan. (B) Experiment 2 was conducted in Ezhou, Hubei. (C) Experiment 3 was conducted in Huashan, Hubei. In experiment 3, V1 stands for Fengliangyou 4, V2 for Luoyou 9348, and V3 for Changjingyou 582. Three levels of nitrogen fertilizer were set up at N1/4 (36 kg/ha), N1 (144 kg/ha), and N2 (288 kg/ha).

**Fig. 2.**
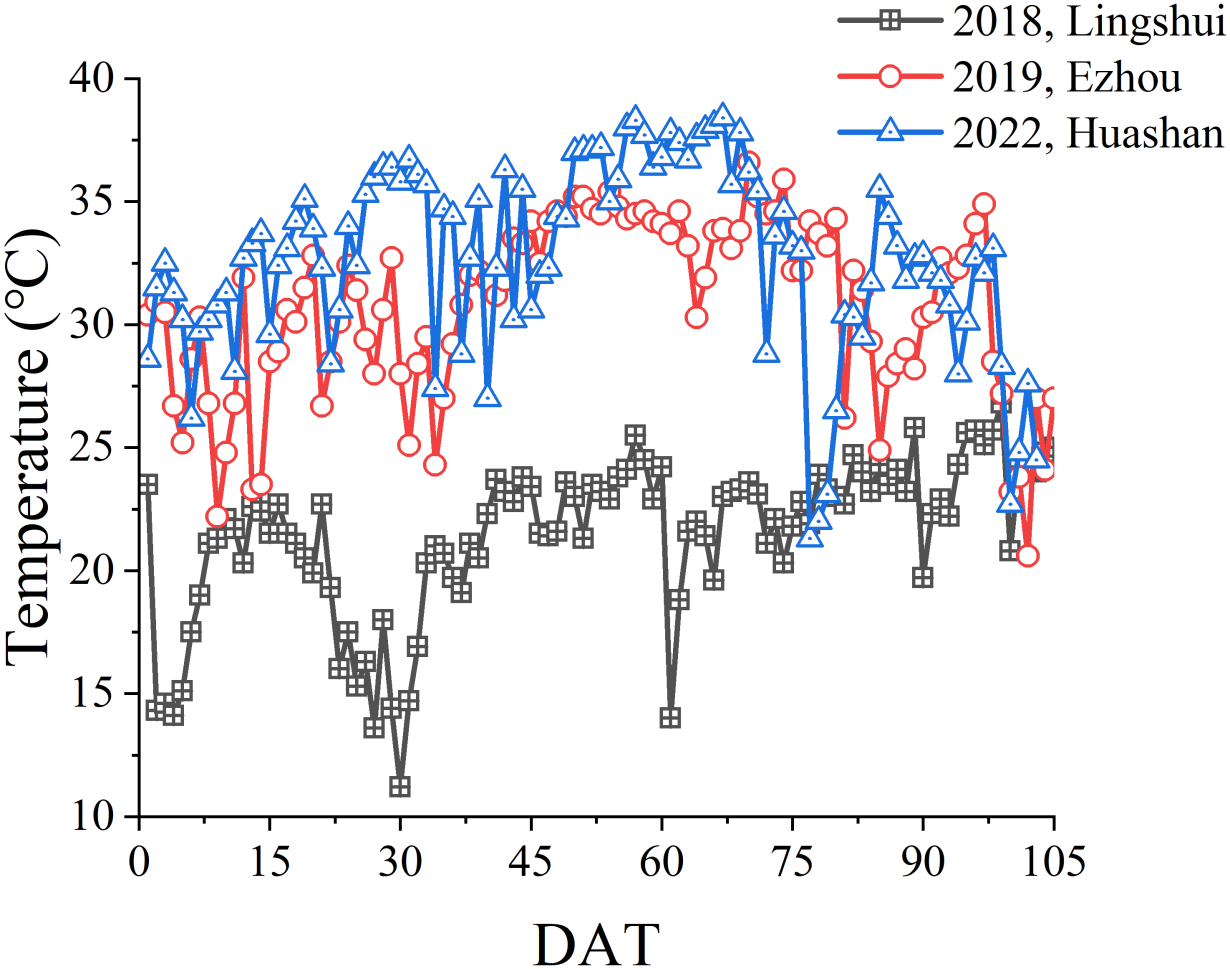
Temperature variations during the rice growth period in Lingshui, Ezhou, and Huashan were recorded by the weather station at 11:00 a.m. every day. The horizontal axis is the days after transplanting (DAT).

This study designed 3 experiments in 3 typical rice planting sites in China, across different years (Fig. 1). The first rice data collection campaign was conducted from November 2017 to May 2018 in Lingshui (18°31' N and 110°3' E), located in Hainan, China. The second data collection was performed from May to September 2019 in Ezhou (30°22' N and 114°44' E), located in Hubei, China. The third data collection was conducted from June to September 2022 in Huashan, located in Hubei, China (30°33' N and 114°31' E). Lingshui is in the southeastern part of Hainan Island. It has a tropical monsoon oceanic climate with abundant rainfall. The annual average rainfall is about 2,100 mm, and the sunshine duration is 2,261.6 h. Ezhou is one of the important cities in the middle reaches of the Yangtze River city group, located in the eastern part of Hubei Province. The climate of the site is subtropical monsoon with an average annual rainfall of 1,282.8 mm and average annual sunshine of 2,003.8 h. Huashan is located in the eastern suburbs of Wuhan City, Hubei Province. The annual rainfall in Wuhan is about 1,300 mm, with rainfall concentrated from June to August every year, and the total annual sunshine hours are about 2,000 h. Huashan has been actively developing in the direction of efficient agriculture in recent years. The temperature profiles when conducting these 3 experiments are plotted in Fig. 2.

The rice varieties selected for experiments 1 and 2 were representative indica varieties from the Yangtze River basin and southern China. A total of 42 cultivars of rice were planted in experiment 1, and a total of 48 cultivars of rice were planted in experiment 2. The transplanting time of rice in the 2-year experiment was 2018 January 8 and 2019 June 9, respectively. Each cultivar was transplanted into a separate plot (Fig. 1). These cultivars applied the same planting density (22.5 bundles/m^2^) and nitrogen fertilizer. The plots in Lingshui and Ezhou were about 63 and 36 m^2^, respectively (Fig. 1). In experiment 1, 12 rows were planted in each plot. In experiment 2, 6 rows were planted. The inter-row spacing was kept at 33 cm and the rows were planted in 2 lines at a spacing of 20 cm.

For the third experiment designed at Huashan in 2022, 3 varieties of rice, namely, Fengliangyou 4 (V1), Luoyou 9348 (V2), and Changjingyou 582 (V3), were selected (Fig. 1, experiment 3). The first 2 varieties were indica rice used in the 2 previous experiments, and the last variety was japonica rice selected to verify the generalization ability of the model. Each variety was repeated on 3 randomly distributed plots. To test the model's generalizability under different nitrogen application rates, in the field, 3 levels of nitrogen fertilizer were set up at N1/4 (36 kg/ha), N1 (144 kg/ha), and N2 (288 kg/ha) (Fig. 1, experiment 3). Approximately 50% of nitrogen fertilizer was utilized as basal fertilizer, 25% of nitrogen fertilizer was applied at the jointing stage, and another 25% of nitrogen fertilizer was applied at the heading stage. The transplanting time of rice was 2022 June 14. Each plot in experiment 3 was about 14 m^2^. These plots applied the same planting density (28 bundles/m^2^).

For the 3 experiments, the data collected from the 2018 Lingshui and 2019 Ezhou experiments were used for modeling, and the 2022 Huashan experiment served as the validation set for model transfer. To test the model more comprehensively, a japonica rice variety was specifically set up at Huashan in 2022 to test the generalizability and robustness of our model.

### Data collection

Six, 13, and 7 field campaigns were conducted in Lingshui, Ezhou, and Huashan, respectively (Table 1). For each campaign, 2 UAVs that carried different sensors were arranged in 2 separate fights to acquire the canopy reflectance and canopy height.

**Table 1.** Data list for the 3 experiments, where the data acquisition time is denoted using DAT.

Time and location	Rice varieties	Data	Data collection dates			
			Tillering	Jointing	Booting and heading	Ripening
2018, Lingshui	42 varieties of indica rice	Image	25,49	62	69	83,97
		LAI and AGB	27,48	60	70	82,99
		Plant height	27,48	60	70	82,99
2019, Ezhou	42 varieties of indica rice	Image	17,23,27,35,43,48	53,58	63,68	74,81,86
		LAI and AGB	17,23,27,37,42,47	53,58	63,69	73,78,85
2022, Huashan	2 varieties of indica rice, 1 variety of japonica rice	Image	18,27,38	49	62	72,83
		LAI and AGB	16,27,38	49	63	72,83

#### UAV-based data collection and processing

The multi-spectral camera (Mini-MCA camera system, Tetracam Inc., Chatsworth, CA, USA), with center bands of 490, 520, 550, 570, 670, 680, 700, 720, 800, 850, 900, and 950 nm, was mounted on an M8 UAV (Beijing TT Aviation Technology Co. Ltd.) to obtain canopy multi-spectral images between 10:00 and 14:00 under clear skies at about 100-m altitudes. At this altitude, the image can completely cover the experimental field, and the resolution of the multispectral image was 5.5 cm/pixel. After shooting, the multi-band images need to be processed by band registration and radiometric calibration. The band registration operation was done in the software PixelWrench2 (Tetracam Inc., Chatsworth, CA, USA). The subband empirical line method was used, and the image digital values were converted to reflectance [11], a correction method suitable for the mini-MCA camera. For image radiometric calibration, 8 blankets with standard reflectance values of 0.03, 0.06, 0.12, 0.24, 0.36, 0.48, 0.56, and 0.80 were laid at the edge of the ground, and the gain and offset values for each band were solved using the least squares method.

A DJ Phantom 4 Pro quadrotor drone (SZ DJI Technology Co. Ltd., Shenzhen, China) was used to acquire RGB aerial images for canopy 3D reconstruction. The drone was equipped with an RGB camera named DJFC 6310. This camera was mounted on the drone body through its gimbal to isolate flight vibrations and ensure that the camera can always be vertically downward during flight. The flight height was set to 30 m with a longitudinal overlap of 90% and a lateral overlap of 70%. The UAV flight speed was 5.4 km/h, and the interval between photos was set to 2 s. The image size was 5,472 × 3,648 pixels and had an image resolution of 0.8 cm/pixel. The images were collected at key periods throughout the rice entire growing season, as shown in Table 1.

After the flight, Agisoft Photoscan Professional software (version 1.4.5, Agisoft LLC, St. Petersburg, Russia) was used to perform 3D point cloud reconstruction to generate digital surface model (DSM). The canopy height (*H*) was calculated by Eq. 1:

$$H=\mathrm{DSM}-{\mathrm{DSM}}_{\text{soil}}$$ (1)

where DSM_soil_ is the DSM of the soil acquired before transplanting.

In each experiment, the average reflectance and average height were obtained by outlining the region of interest (ROI) of each plot. Ten plot-level VIs were constructed from the plot-level canopy reflectance according to the equations in Table 2. These VIs have been found to be effective in estimating crop growth parameters [16,23,26,51–53] and consist of different bands and different combinations, including ratio-based VIs (CI_green_ and CI_red edge_), normalized VIs (NDVI, NDRE, and GNDVI), and VIs commonly used to estimate rice growth parameters in recent years (MTCI, WDRVI, OSAVI, and EVI2).

**Table 2.** The vegetation indices used in the study and the formulas used to calculate them.

VI	Formula	Reference
Green-edge chlorophyll index (CI_green_)	*R*_800_/*R*_550_ − 1	[82]
Red-edge chlorophyll index (CI_red edge_)	*R*_800_/*R*_720_ − 1	[82]
Green normalized difference vegetation index (GNDVI)	(*R*_800_ − *R*_550_)/(*R*_800_ + *R*_550_)	[64]
Normalized difference vegetation index (NDVI)	(*R*_800_ − *R*_670_)/(*R*_800_ + *R*_670_)	[83]
Normalized difference red edge (NDRE)	(*R*_800_ − *R*_720_)/(*R*_800_ + *R*_720_)	[84]
MERIS terrestrial chlorophyll index (MTCI)	(*R*_800_ − *R*_720_)/(*R*_720_ − *R*_670_)	[85]
Wide dynamic range vegetation index (WDRVI)	(*α* × *R*_800_ − *R*_670_)/(*α* × *R*_800_ + *R*_670_), *α* = 0.2	[23]
Optimized soil-adjusted vegetation index (OSAVI)	(1+0.16)×(*R*_800_ − *R*_720_)/(*R*_800_ + *R*_720_ + 0.16)	[86]
Two-band enhanced vegetation index (EVI2)	2.5 × (*R*_800_ − *R*_670_)/(1 + *R*_800_ + 2.4 × *R*_670_)	[31]

#### Destructive measurements of LAI and AGB

LAI of rice was acquired through an LI-3100C leaf area meter (LI-COR, Lincoln, NE, USA). In each campaign, 3 bunches of rice plants randomly selected in each plot were collected, placed in muddy water, and then transported to the laboratory. After removing the root, the remaining part was divided into green leaves, dead leaves, stems, and panicles. The green leaves were put into the leaf area meter, and LAI of each plot was calculated by density. The collected leaves, stems, and panicles were placed in an oven and dried first at 105 °C for 30 min and then adjusted to dry at 80 °C until the weight was constant, and the dry AGB was obtained by weighing. There were 252, 624, and 189 samples collected in Lingshui, Ezhou, and Huashan, respectively. The data collected in Lingshui in 2018 and Ezhou in 2019 were used for data analysis and model building, and the data collected from Huashan in 2022 were used to validate the model.

#### Measurements of plant height

To verify whether the canopy height extracted from UAV imagery can replace the height of the manual measurement, we measured rice plant height in the experiment conducted in 2018 in Lingshui County, Hainan. For each plot, 3 bundles of rice were randomly selected for measurement. The height from the ground to the highest part of the rice plant was measured, and the average plant height of these 3 bundles was calculated and recorded as the average plant height of the plot.

### Model development

#### Modeling of LAI based on the vertical distribution of rice

LAI is the vertical integral of the leaf area density (LAD) [54]. LAD is considered as the leaf area of one side per unit of horizontal layer volume [55]. LAI is obtained from the vertical integration of the LAD(*z*) values.

$$\mathrm{LAI}=\int_{0}^{H}\text{LAD}\left(z\right)\;\mathrm{dz}$$ (2)

where the height of the canopy is *H* and the integral variable is z.

**Fig. 3.**
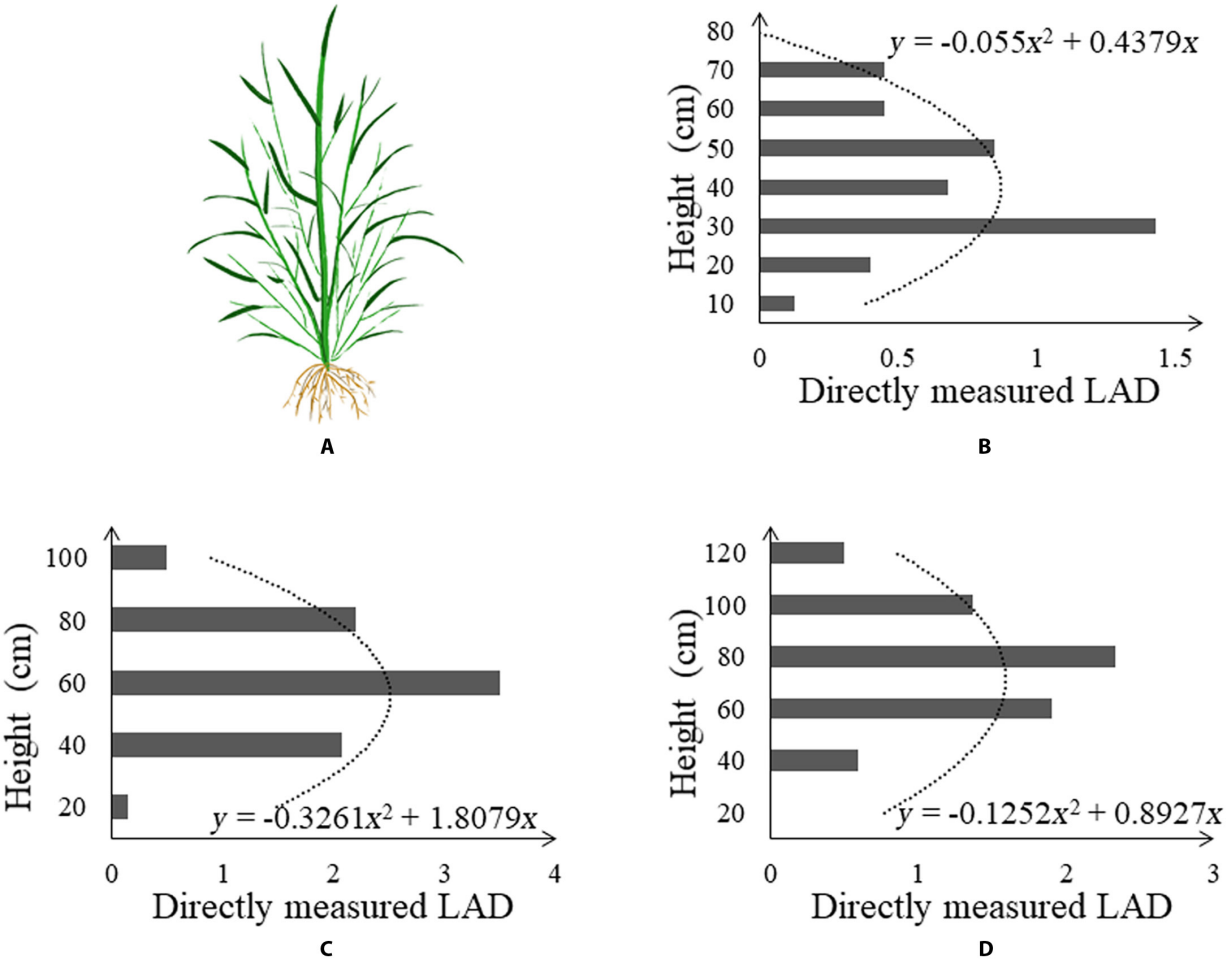
(A) Pattern illustration of an individual rice plant. The directly measured LAD at (B) tillering stage, (C) booting stage, and (D) ripening stage.

As shown in Fig. 3, the vertical distribution of the rice LAI is large in the middle and small at both ends. This vertical morphological feature of rice has also been described by Wang et al. [56], Hirooka et al. [57], Burgess et al. [58], and Guo et al. [59]. For rice plant throughout the entire growing season, its vertical distribution is described by a quadratic equation (Fig. 3B to D), which are then solved:

$$\mathrm{LAD}\left(z\right)=a_{1}\cdot z^{2}+a_{2}\cdot z$$ (3)

where *a*_1_ and *a*_2_ are 2 coefficients and *z* represents the height from the ground.

When assuming that the maximum value of LAD (LAD_max_) is obtained at half height, LAI is given by:

$$\mathrm{LAI}=\int_{0}^{H}\text{LAD}\left(z\right)\;\mathrm{dz}=\frac{2}{3}\cdot {\mathrm{LAD}}_{\max}\cdot H$$ (4)

where the height of the canopy is *H*.

If the rice canopy is projected onto the image plane, the maximum LAD is strongly correlated with the vegetation cover [56]. A large amount of literature shows that VI can estimate vegetation cover [15,17,60], so the VI can be used to characterize the maximum LAD. Equation 4 can be easily converted to:

$$\mathrm{LAI}\propto \left(\mathrm{VI}\cdot H\right)$$ (5)

Therefore, the VI calculated from multispectral images and height information derived from the 3D modeling are the key parameters for calculating LAI.

#### Modeling of aboveground biomass based on rice photosynthetic accumulation

Photosynthesis is a process of absorbing and converting light energy into chemical energy stored in the form of carbohydrates. Most of the dry matter of green plants (about 80 to 90%) comes from photosynthesis, while the rest comes from the soil [61]. The dry matter production of green plants is a gradual and accumulative process and is primarily determined by photosynthetic efficiency, LAI, and the duration time during which photosynthesis continues [45]. Most of the biomass of rice is concentrated in the aboveground part [62]. The “net assimilation rate” (NAR) is a useful indicator of green plants' photosynthetic efficiency. It is defined as the rate of increase in dry weight per unit of leaf area [45,63]. Hence, the relationship between aboveground dry biomass, NAR, and LAI is given by:

$$\frac{\mathrm{dW}}{\mathrm{dt}}=\mathrm{NAR}\cdot \mathrm{LAI}$$

$$W=\int_{0}^{t}\mathrm{NAR}\cdot \mathrm{LAI}\cdot \mathrm{dt}$$

$$W\approx \sum_{i=1}^{n}{\mathrm{NAR}}_{i}\cdot {\mathrm{LAI}}_{i}\cdot \left(t_{i}-t_{i-1}\right)$$ (6)

where *W* is the aboveground dry biomass. *t* denotes the day after transplanting. *t_i_* and *t*_*i* − 1_ denote the days after the *i*th and (*i* − 1)th transplanting, respectively.

Remote sensing methods have been proven to be able to estimate the photosynthetic capacity of green vegetation. Numerous studies have proven that the VI can reflect the chlorophyll content of green vegetation and has a strong correlation with the photosynthetic capacity of vegetation [20,64,65]. Combined with the LAI model proposed in the previous section, we propose a PAM based on the accumulation to calculate aboveground dry biomass (Eq. 7).

$$W\propto \sum_{i=1}^{n}{\mathrm{VI}}_{i}\cdot {\mathrm{LAI}}_{i}\cdot \left(t_{i}-t_{i-1}\right)$$

$$W\propto \sum_{i=1}^{n}{{\mathrm{VI}}_{1}}_{i}\cdot {{\mathrm{VI}}_{2}}_{i}\cdot h_{i}\cdot \left(t_{i}-t_{i-1}\right)$$ (7)

where *VI*_1*i*_ and *VI*_2*i*_ are 2 VIs obtained at the *i*th remote sensing observation, respectively, and *h_i_* is the canopy height obtained at the *i*th remote sensing observation.

#### Simplification of PAM model based on time-series analysis

By analyzing the time-series accumulative trend, we plan to design a more general and efficient model to estimate AGB that reduces the number of flights and observations.

**Fig. 4.**
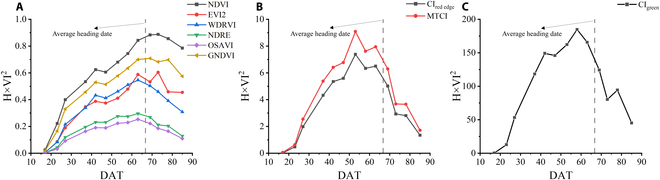
Temporal behaviors of average H × VI^2^ during the entire growing season in 2019. Three groups were classified by the value range of H × VI^2^, where (A) indicates the results when selecting NDVI, EVI2, WDRVI, NDRE, OSAVI, and GNDVI, (B) indicates the results when selecting CI_red edge_ and MTCI, and (C) indicates the results when selecting CI_green_.

The temporal behavior of the average H × VI^2^ throughout the growing season is divided into 3 groups according to the range of values (Fig. 4). Three groups were classified by the value range of H × VI^2^, where (A) indicates the results when selecting NDVI, EVI2, WDRVI, NDRE, OSAVI, and GNDVI, (B) indicates the results when selecting CI_red edge_ and MTCI, and (C) indicates the results when selecting CI_green_. From the time the season begins, the value rose to the highest value and then decreased. If we now turn to Fig. 4A, it is apparent that the heading is the key stage for AGB estimation throughout the growing season using PAM of these 6 VIs.

**Fig. 5.**
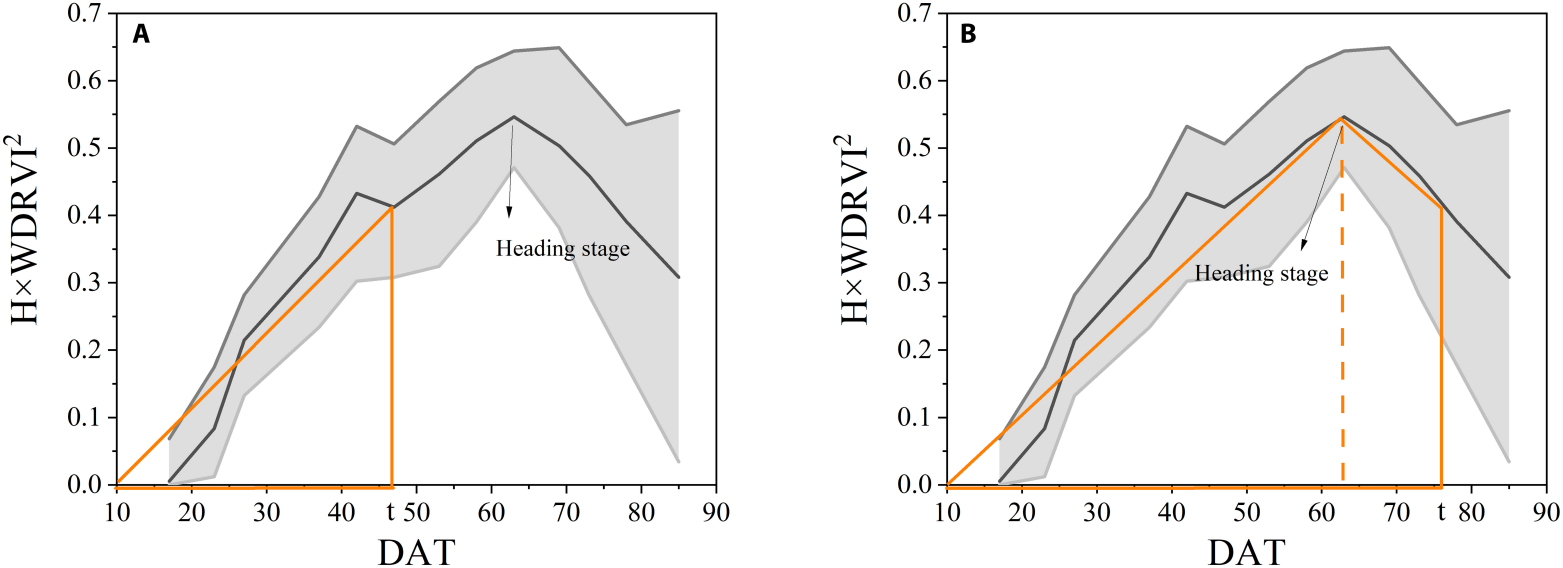
Taking H × WDRVI^2^ as an example, the illustration of the piecewise simplified model (A) when the period to be estimated is earlier than the heading stage and (B) when the period to be estimated is later than the heading stage. The horizontal axis is DAT.

A piecewise SPAM such as Eq. 8 is proposed to estimate the full growth period AGB by sampling the heading stage, where VI can be selected from NDVI, EVI2, WDRVI, NDRE, OSAVI, and GNDVI. Taking WDRVI as an example, the estimated AGB before the pre-heading stage can be obtained by directly calculating the area of the triangle (Fig. 5A), while the estimated AGB after the post-heading stage can be calculated by adding the area of the triangle before heading and the area of the trapezoid after the heading stage (Fig. 5B).

$$\begin{array}{c} W\propto \left\{\begin{array}{c} \frac{{\mathrm{VI}}^{2}\cdot H\cdot \left(t-t_{0}\right)}{2},\;t\leq T_{1}\\ \frac{{\mathrm{VI}}_{T1}^{2}\cdot H_{T1}\cdot \left(T_{1}-t_{0}\right)}{2}+\frac{\left({\mathrm{VI}}^{2}\cdot H+{\mathrm{VI}}_{T1}^{2}\cdot H_{T1}\right)\cdot \left(t-T_{1}\right)}{2},\;t>T_{1} \end{array}\right. \end{array}$$ (8)

where *T*_1_ represents DAT (days after transplanting) of the heading stage. VI can be selected from NDVI, EVI2, WDRVI, NDRE, OSAVI, and GNDVI.

#### Model accuracy evaluation

The results were evaluated using the coefficient of determination (*R*^2^), root mean square error (RMSE), and relative root mean square error (rRMSE) [5,8,65,66]. The formulas are shown in Eqs. 9 to 11. The closer *R*^2^ is to 1, the smaller the RMSE and rRMSE, representing the higher accuracy of the model in predicting growth parameters. To test the transferability and generalizability of the model, the data collected from experiments 1 and 2 were used for modeling, and the data from experiment 3 were tested.

$$R^{2}=\frac{\sum_{i=1}^{n}{\left({\hat{y}}_{i}-\bar{y}\right)}^{2}}{\sum_{i=1}^{n}{\left(y_{i}-\bar{y}\right)}^{2}}$$ (9)

$$\text{RMSE}=\sqrt{\frac{\sum_{i=1}^{n}{\left(y_{i}-{\hat{y}}_{i}\right)}^{2}}{n}}$$ (10)

$$\text{rRMSE}=\frac{\text{RMSE}}{\bar{y}}\times 100\%$$ (11)

where *y_i_*, $\;{\hat{y}}_{i}$, and $\bar{y}$ represent the measured value, predicted value, and mean value, respectively, and *n* is the number of samples.

## Results

### Verification of the accuracy of canopy height estimated by remote sensing

**Fig. 6.**
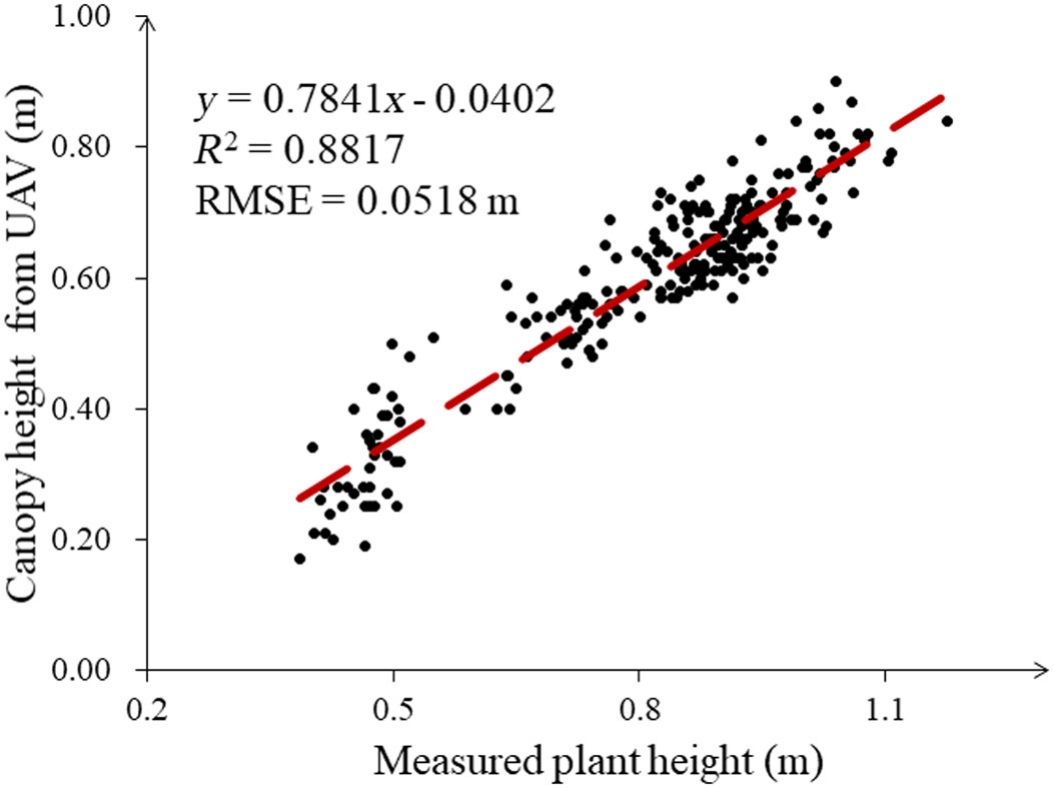
Relationship between canopy height derived from UAVs and the measured plant height at Lingshui in 2018.

Our experiments in all 3 years were measured at critical periods throughout the growing season (Table 1), and the accuracy of DSM was the same in all 3 experiments with the same instrumentation and consistent flight parameters. Remote sensing methods for obtaining canopy height have been widely used in previous studies [5,32,67–70], and we validated the accuracy of this method throughout the rice growing season in experiment 1. Figure 6 shows the correlation between the average canopy height calculated from the UAV images using Eq. 1 and the actual collected rice plant height in experiment 1. The measured plant heights were generally higher than those obtained by remote sensing, which is because the measured plant heights were measured after the tallest leaves were stretched straight. However, even so, *R*^2^ between remotely sensed canopy height and measured height was 0.88 and the RMSE was 0.05 m, showing a good correlation. Therefore, canopy height calculated by remote sensing can be used as a substitute for plant height in subsequent experiments for analysis and application.

### LAI estimation based on the vertical distribution model of rice

**Fig. 7.**
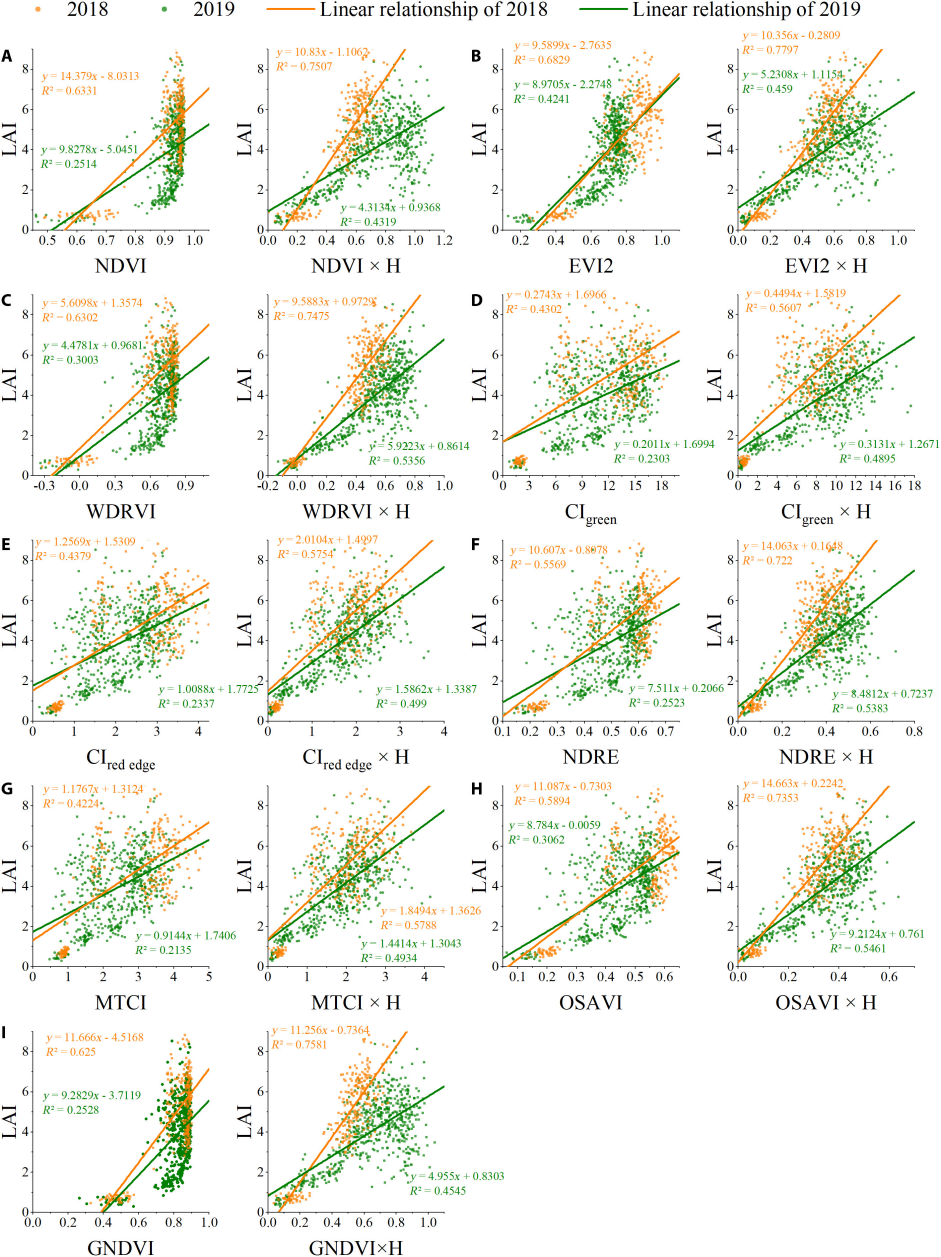
Estimation of LAI of rice during the whole growing period by LAI versus VI and LAI versus (VI × H) models in 2018 and 2019 based on (A) NDVI, (B) EVI2, (C) WDRVI, (D) CI_green_, (E) CI_red edge_, (F) NDRE, (G) MTCI, (H) OSAVI, and (I) GNDVI.

**Fig. 8.**
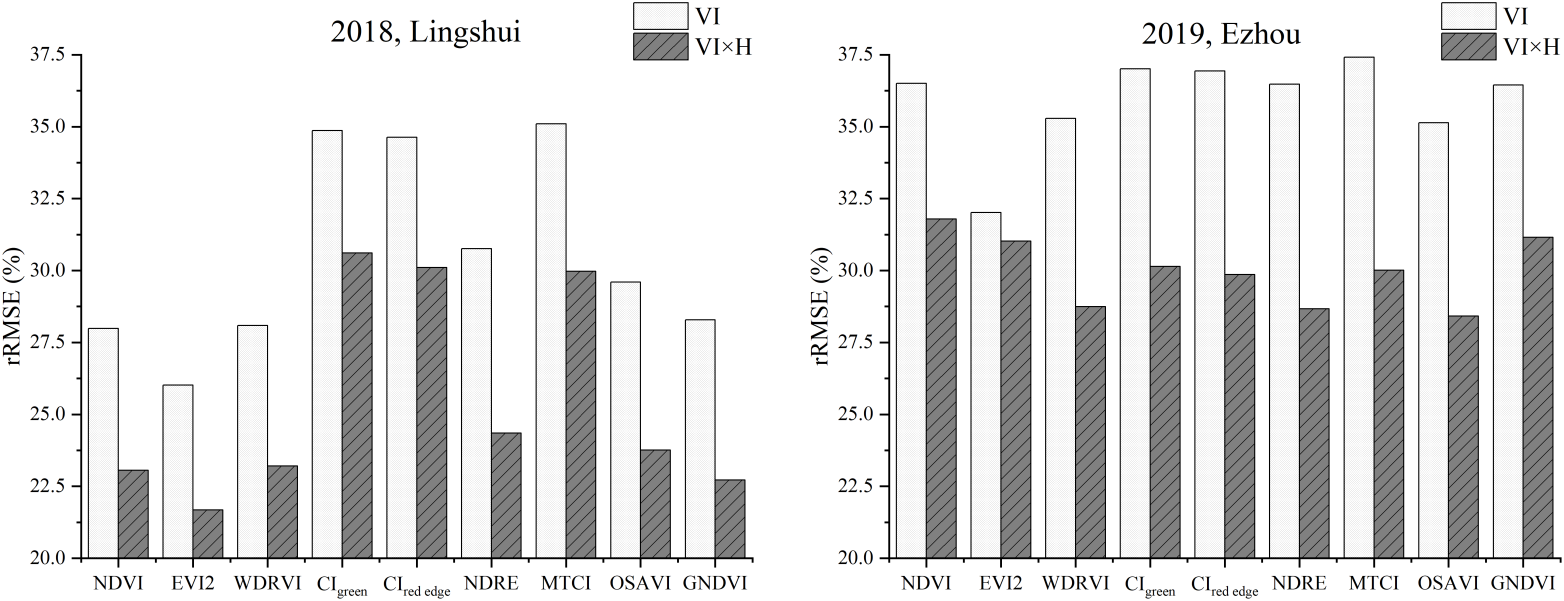
The rRMSE of LAI versus VI and LAI versus (VI × H) during the whole growing period in 2018 and 2019 for 9 tested indices.

Nine VIs were tested to compare the relationship between LAI versus VI and LAI versus H × VI. The scatter diagram and regression analysis of these VIs within 2 years were shown in Fig. 7, and the summary statistics of all 9 VIs were presented in Table 3. The sample data in 2018 are sparser than those in 2019 (Fig. 7), and *R*^2^ is also higher than that in 2019 (Fig. 7 and Table 3), while their RMSE is relatively similar, and the difference is less than 0.2 (Table 3). The samples are distributed relatively discretely in the ratio VI (CI_green_ and CI_red edge_) and MTCI, and *R*^2^ of these VIs is also lower than that of the other VIs (Table 3). From the VI model in Fig. 7, it is apparent that the model at medium and high LAI values (LAI > 4) is easily saturated. For the data acquired in 2019, dense observations can also show the hysteresis effect reported in [71]. Turning to the experimental evidence for the H × VI model, it is found that this saturation and hysteresis effect was greatly reduced (Fig. 7). Further statistical tests on 9 VIs showed that the H × VI model all had a greater ability to estimate LAI than VI models (Table 3). For the other 8 VIs except for EVI2, rRMSE can be improved by more than 4% in both years (Fig. 8). From Fig. 8, for NDRE, more than 6% improvement results are obtained in both years of experiments.

**Table 3.** Comparison of LAI versus VI and LAI versus (VI × H) during the whole growing period in 2018 and 2019 for 9 tested indices.

VI	2018, Lingshui				2019, Ezhou			
	**VI**		VI × H		VI		VI × H	
	*R* ^2^	RMSE	*R* ^2^	RMSE	*R* ^2^	RMSE	*R* ^2^	RMSE
NDVI	0.633	1.323	0.751	1.090	0.251	1.440	0.432	1.254
EVI2	0.683	1.230	0.780	1.025	0.424	1.263	0.459	1.224
WDRVI	0.630	1.328	0.748	1.097	0.300	1.392	0.536	1.134
CI_green_	0.430	1.648	0.561	1.447	0.230	1.460	0.490	1.189
CI_rededge_	0.438	1.637	0.575	1.423	0.234	1.457	0.499	1.178
NDRE	0.557	1.454	0.722	1.151	0.252	1.439	0.538	1.131
MTCI	0.422	1.659	0.579	1.417	0.214	1.476	0.493	1.184
OSAVI	0.589	1.399	0.735	1.123	0.306	1.386	0.546	1.121
GNDVI	0.625	1.337	0.758	1.074	0.253	1.438	0.455	1.229

### Biomass estimation based on rice PAM

**Fig. 9.**
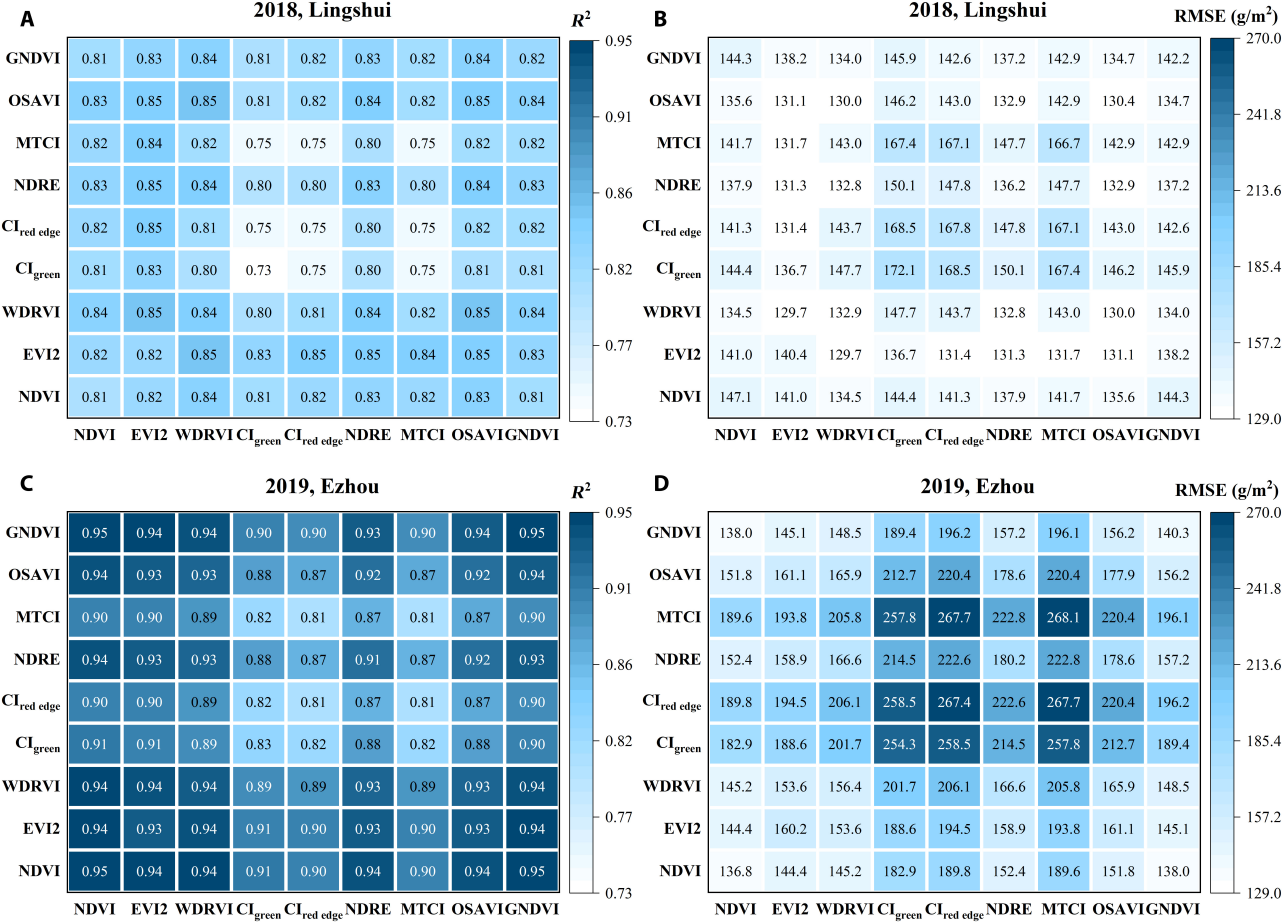
*R*^2^ and RMSE of the simple linear regression models based on $\sum_{i=1}^{n}{\mathrm{VI}}_{1_{i}}\cdot {\mathrm{VI}}_{2_{i}}\cdot h_{i}\cdot \left(t_{i}-t_{i-1}\right)$ for rice AGB estimation during the growing season of 2018, Lingshui and 2019, Ezhou. (A) and (B) are *R*^2^ and RMSE of Lingshui in 2018, respectively. (C) and (D) are *R*^2^ and RMSE of Ezhou in 2019, respectively. The *x* axis and *y* axis represent the VI selections of VI_1_ and VI_2_ for AGB calculation, respectively.

According to Eq. 7, 2 VIs with different meanings are used in PAM. Figure 9 compares *R*^2^ and RMSE using different VI combinations in PAM. Similar to the results for estimating LAI, *R*^2^ and RMSE on the ratio VI (CI_green_ and CI_red edge_) and MTCI are also worse than the other VIs. A closer inspection of the figure showed that any 2 VIs from NDVI, EVI2, WDRVI, NDRE, OSAVI, and GNDVI were put into the PAM model, and stable and good results were obtained in the experiments over 2 years. In addition, *R*^2^ and RMSE using themselves in the model twice are great (on the diagonal of each matrix through the origin). Therefore, we can get good results by selecting one of the 6 excellent VIs and using it twice in the model.

**Fig. 10.**
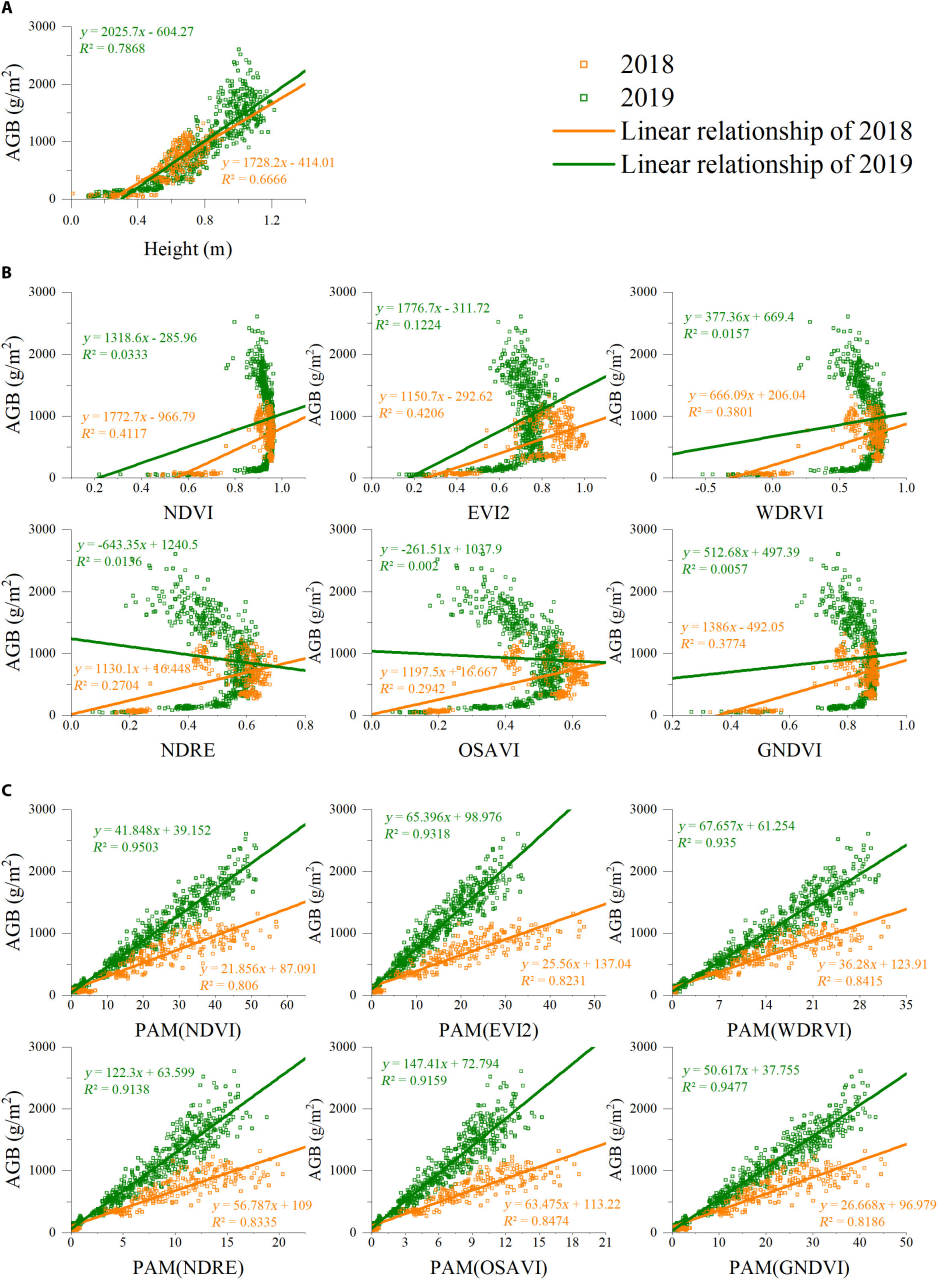
The variation of AGB plotted with (A) height, (B) VI, and (C) PAM of rice throughout the growing seasons in 2018 and 2019. The same 2 VIs were used in PAM.

Figure 10 presents the variation of height, VI, and PAMs plotted with AGB throughout the season. The best 6 VIs in estimating rice AGB and PAMs using these 6 VIs were selected for display. The average value of AGB in 2018 is smaller than that in 2019. In 2018, the maximum value of AGB does not exceed 1,500 g/m^2^, while the maximum value in 2019 even exceeds 2,500 g/m^2^ (Fig. 10). The differences in AGB between the 2 years may be caused by different regions, seasons, and temperatures. With successive increases of the rice AGB, the height of rice also increased (Fig. 10A), but VI increased first and then decreased (Fig. 10B), which greatly affected the ability of VI to estimate AGB in the whole growth period. However, the increase of AGB with height is not completely linear, which makes *R*^2^ also limited. The result obtained from PAM is set out in Fig. 10C. It is apparent from this figure that PAM is very effective for AGB estimation. There was a remarkable positive correlation between PAM and AGB. In the 2-year experiment, *R*^2^ exceeded 0.8. Further analysis showed that the slope of the fitting line in 2 years is different. The slope of the 2018 data is larger than that of the 2019 data, that is, with the same increase in AGB, the PAM change in 2018 is larger than that in 2019. This may be related to the different planting locations, planting times, and temperatures in the 2 years.

Table 4 provides the summary results for all tested VIs, height, and PAM. The same 2 VIs were used in PAM. EVI2 was the best VI model for estimating AGB; however, *R*^2^ was still below 0.5 in 2 years of experiments. It can be seen in this table that PAM achieves much better results than the VI and height models. For the VI model in 2018, PAM's *R*^2^ increased by more than 0.39, and the RMSE decreased by more than 100 g/m^2^. For the VI model in 2019, PAM's *R*^2^ increased by more than 0.7, and the RMSE decreased by more than 300 g/m^2^. For the height model, *R*^2^ of PAM with NDVI, EVI2, WDRVI, NDRE, OSAVI, and GNDVI increased by more than 0.13 in both 2018 and 2019. The RMSE of PAM with these VIs can be reduced by 45 and 100 g/m^2^ in 2018 and 2019, respectively.

**Table 4.** The results of VI model and PAM were compared through 2 years of experiments (the same 2 vegetation indices were used in PAM).

	2018, Lingshui						2019, Ezhou					
VI	VI model		H model		PAM		VI model		H model		PAM	
	*R* ^2^	RMSE (g/m^2^)	*R* ^2^	RMSE (g/m^2^)	*R* ^2^	RMSE (g/m^2^)	*R* ^2^	RMSE (g/m^2^)	*R* ^2^	RMSE (g/m^2^)	*R* ^2^	RMSE(g/m^2^)
NDVI	0.41	256.08	0.67	192.79	0.81	147.06	0.03	603.33	0.79	283.33	0.95	136.81
EVI2	0.42	254.14			0.82	140.41	0.12	574.86			0.93	160.22
WDRVI	0.38	262.87			0.84	132.94	0.02	608.80			0.94	156.40
CI_green_	0.17	304.09			0.73	172.14	0.01	609.43			0.83	254.29
CI_red edge_	0.17	303.92			0.75	167.79	0.03	605.25			0.81	267.36
NDRE	0.27	285.17			0.83	136.23	0.01	609.43			0.91	180.16
MTCI	0.16	306.03			0.75	166.65	0.04	602.34			0.81	268.13
OSAVI	0.29	280.48			0.85	130.42	0.00	613.01			0.92	177.90
GNDVI	0.38	263.44			0.82	142.19	0.01	611.88			0.95	140.26

### Biomass estimation based on rice SPAM

**Fig. 11.**
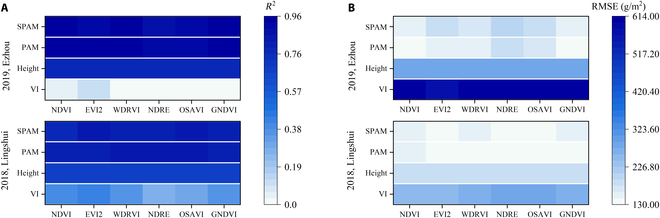
(A) *R*^2^ and (B) RMSE of AGB estimation throughout the entire growing season were calculated based on the VI model, height model, PAM, and SPAM in 2018, Lingshui, and 2019, Ezhou.

**Fig. 12.**
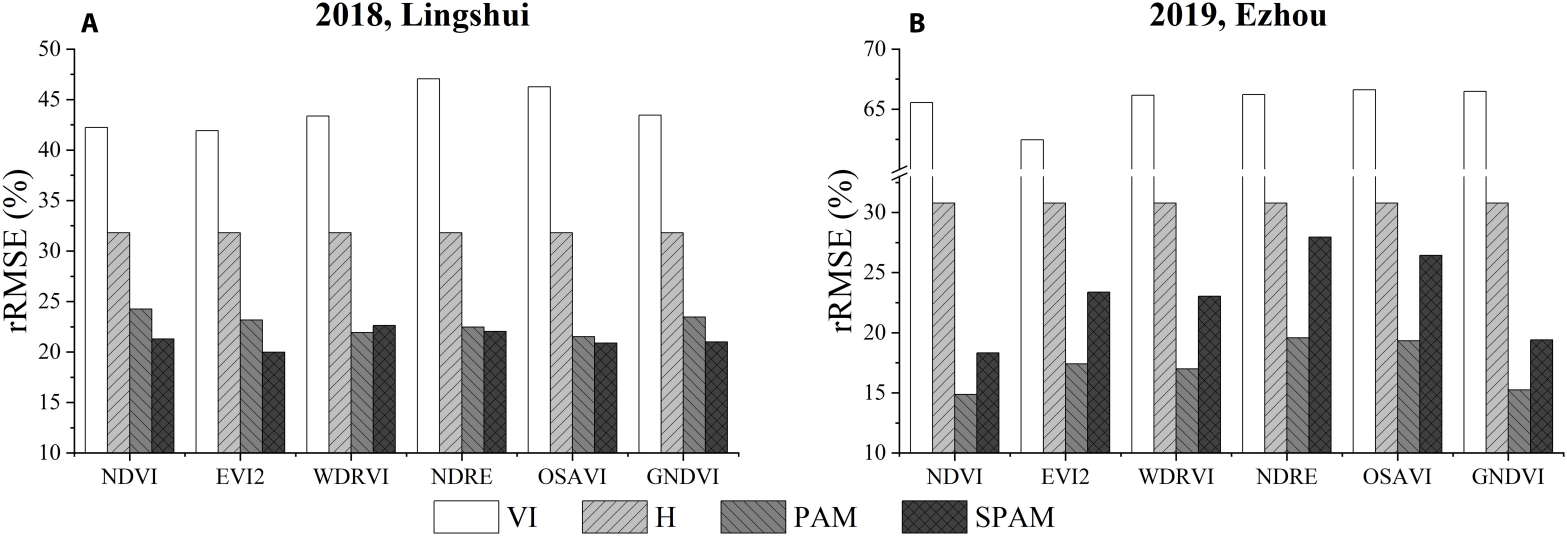
rRMSE of AGB estimation throughout the entire growing season was calculated based on the VI model, height model, PAM, and SPAM in (A) 2018, Lingshui, and (B) 2019, Ezhou.

The results of *R*^2^ and RMSE using the VI model, height model, PAM, and SPAM were shown in Fig. 11. In the 2-year experiment, VI had the lowest *R*^2^ and the highest RMSE, followed by the height model. The values of *R*^2^ and RMSE were similar for PAM and SPAM, and SPAM was slightly lower than PAM. The rRMSE results of 4 different models were shown in Fig. 12. The rRMSE of SPAM is slightly higher than PAM but much lower than the VI and H models. Compared with height models, SPAM can improve the accuracy of the estimation, where rRMSE decreased by 6.65 to 9.39% in 2018 and by 8.46 to 14.75% in 2019 (Fig. 12).

### Model transferability

**Fig. 13.**
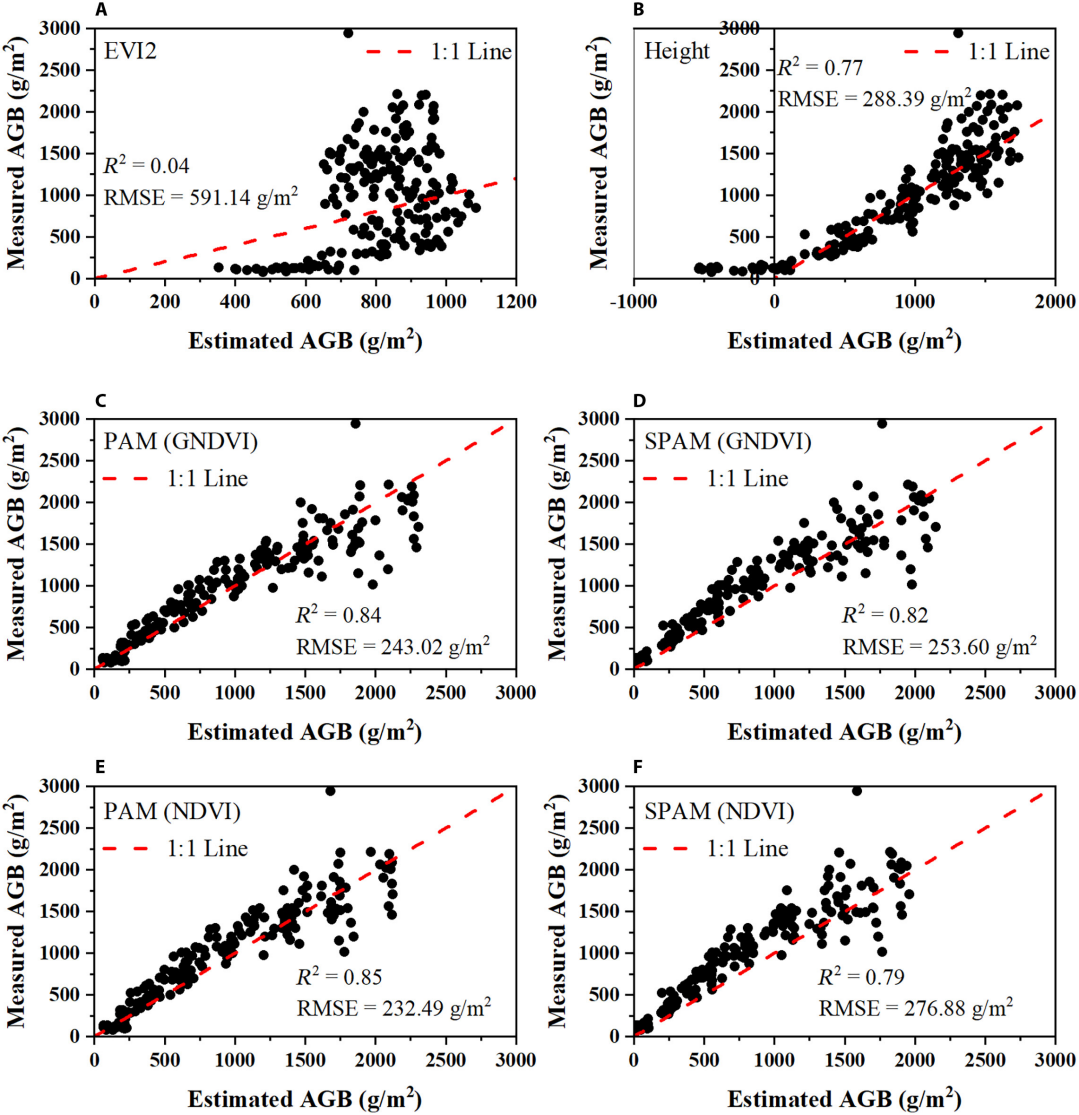
Measured versus estimated aboveground biomass (g/m^2^) of 2022 samples predicted by the model established in 2018 and 2019 samples based on (A) EVI2 model, (B) height model, (C) PAM using GNDVI, (D) SPAM using GNDVI, (E) PAM using NDVI, and (F) SPAM using NDVI. Red dashed lines indicate the expected 1:1 relationship.

The models using GNDVI and NDVI were the best at estimating AGB in various rice cultivars throughout the entire growing season (Fig. 12). These models were applied to test the data collected at Huashan in 2022. The results of these models compared to the best VI model (EVI2) and height model are presented in Fig. 13.

It can be seen that the AGB model predicted by the VI model is invalid, especially at AGB > 800 g/m^2^. While using height directly for the prediction of AGB of rice throughout the growing season, there are negative values in the early stage (AGB < 250 g/m^2^), which is not consistent with the objective facts. However, the AGB estimated by the PAM and SPAM models exhibited a superior linear relationship with the measured AGB, which was better than the VI and height models. *R*^2^ of the PAM model is above 0.8 regardless of whether the input parameter is GNDVI (Fig. 13C) or NDVI (Fig. 13E), showing a very high superiority compared to the VI and height models. The SPAM model shows a slight decrease compared to the PAM model. These results suggest that SPAM still achieves better results than traditional methods for estimating AGB, and the loss of accuracy compared to PAM is acceptable given the smaller observational workload. If high accuracy is required, especially in the case of low AGB values (<1,000 g/m^2^), it is recommended to use PAM for rice AGB estimation.

## Discussion

As mentioned in the literature review, the spectral VI is the most widely used model to estimate crop AGB [72–74]. In this study, rice AGB versus VI (Fig. 10B) showed that with the rapid growth of rice and the closure of the canopy, the VI growth rapidly reaches a high value and saturates. Then, the VI begins to decrease as the panicle emerged from the leaf sheath, with a significant hysteresis of AGB versus VI relationships before and after panicle emergence. Several recent kinds of literature found that crop height achieved better results than VI in crop AGB estimation [29,70,75,76]. But it can be found in the 2 years of experiments that the height rises rapidly with the increase of AGB, and then spreads out slightly, so the estimation accuracy is also limited (Fig. 10A).

In this study, PAM was proposed to increase the precision of AGB estimation over the whole growing season. This model was guided by the fundamental principle of plant photosynthesis, that is, the accumulation of dry matter resulting from the accumulation of photosynthesis over a period of time on all leaves of the plant [43,77–79]. Since LAI refers to a variable that makes up the accumulative model (Eq. 7), the use of remote sensing data to estimate LAI is important for predicting AGB. An increasing number of researches have revealed that the vertical distribution of canopy biochemical parameters (e.g., LAI, chlorophyll, and nitrogen) is heterogeneous within the vegetation canopy [80]. For the rice canopy, we measured the vertical distribution of the leaf area and acquired a bell-shaped distribution with a large in the middle and a small at both ends (Fig. 3). This bell-shaped distribution observed in this study has also been reported by Hosoi and Omasa [55] and Wang et al. [56], who measured the rice leaf area distribution with a portable scanning lidar and a leaf area meter, respectively. Based on the above analysis, a quadratic equation was used to describe the bell-shaped distribution and obtained a relationship between LAI and H × VI (Eqs. 3 to 5). This also accords with our earlier research, which found that the H × VI model outperformed the VI model in LAI estimation throughout the entire growing season [50]. But earlier studies only demonstrated the complementary effect of height on VI using 1-year data. In this study, the model was constructed from a bell-shaped distribution and tested in 2-year data, which is shown in Fig. 7 and Table 3, presenting this combination as more reliable and robust. Another variable in PAM is NAR, which is an indicator of the photosynthetic capacity of the canopy [44,58,63]. The photosynthetic capacity of vegetation is directly correlated with chlorophyll content. Most of the VIs chosen in this paper cover the 550- and 670-nm bands that reflect the absorption and reflection of light by chlorophyll, and these VIs are strongly correlated with chlorophyll content [44,81]. Therefore, VI was used to replace NAR here. Then, combined with the process of vegetation photosynthesis, SPAM for rice AGB estimation during the whole period with only remote sensing parameters was established.

The PAM achieves a very high accuracy result, with an *R*^2^ higher than 0.7 for the 2018 data and 0.8 for the 2019 data (Table 4). Regression analysis revealed that the results from PAM are more linear than the VI models and H model (Fig. 10). Although the relationship between these 2 indices themselves and AGB saturates after the heading stage, the accumulation of H × VI^2^ is increasing with crop growth and does not saturate. Therefore, this saturation did not occur by considering the AGB photosynthesis accumulation process for modeling (Fig. 10C). When using different VIs, the results of using the PAM model to estimate the AGB over the entire growing season varied significantly (Table 4 and Fig. 9). If PAM selected NDVI, EVI2, WDRVI, NDRE, OSAVI, and GNDVI, the best and most stable results were obtained in the 2 years of the experiment (Figs. 9 and 10C). These VIs had the commonality that H × VI^2^ was increasing before heading and decreased slightly after heading (Fig. 4A). Although PAM obtained exciting and excellent results, it must be observed at high frequencies in that year of the experiment. Thus, based on this temporal behavior of average H × VI^2^ during the entire growing season in 2019 (Fig. 4A), a simplified algorithm is proposed for AGB estimation through limited observations (Eq. 8). After 2 years of experiments, the stability of this SPAM is also proved (Figs. 11 and 12). It can thus be suggested that if we need high accuracy of AGB estimation for the entire growing season, PAM and multiple observations can be used. If we want to reduce the number of observations, SPAM is more suitable for the needs.

To further verify the transferability of PAM and SPAM at different locations in different years, we conducted an experiment specifically in 2022. A total of 851 samples of data collected from 2018 and 2019 were used as the training set to build the model, and a total of 189 samples collected in 2022 were used to test the model. Compared to the VI and height models, PAM and SPAM transfer better and maintain better linear relationships with a stronger ability to estimate AGB throughout the growing season (Fig. 13). The PAM and SPAM had better robustness and generalizability, making it possible to estimate rice AGB across years. In future research, the effects of different environmental conditions on the model can be investigated more thoroughly to better improve the transferability of the model.

The algorithms used in this study are linear regressions that worked efficiently, so we do not need to go for sophisticated algorithms (e.g., machine learning method) with big computation and requiring large amounts of data, which are sometimes costly or unavailable. Comparatively, our proposed algorithm requires a much lower amount of data, especially the SPAM algorithm proposed in this study, which requires observation modeling in the current and heading stages, with slightly lower model accuracy than PAM, but with much lower observation frequency. The UAV platform can easily acquire the spectral information and height information of the canopy. Thus, our proposed method can easily accomplish the monitoring and estimation of AGB for the whole growing period of rice. This method provides a quantitative means to evaluate the growth status of rice with a simple and efficient operation and has the potential to be used on a large scale.

## Conclusion

In this study, AGB estimation of different rice cultivars throughout the growing season was investigated remotely using VI and height derived from UAVs. High-resolution digital and multispectral images of multi-cultivar rice through the UAV platform were acquired. These tested VIs were calculated from the canopy reflectance, and the canopy height was obtained from the RGB images. The LAI estimation model based on the vertical distribution of rice was developed to achieve a high-accuracy prediction of LAI of rice. Due to the saturation of VI during the canopy closure and the decrease of VI after the emergence of rice panicles, the correlation between VIs and AGB was very low during the whole season. The correlation between canopy height and AGB is stronger than that with VI during the rice whole growth period, and *R*^2^ has reached more than 0.6 in the 2 years' data. However, the rapid growth at tillering stage makes this relationship nonlinear and affects the accuracy. Considering the accumulation of AGB, a PAM was constructed by combining height and VI based on the LAI estimation and photosynthesis. To reduce the observations, a simplified version of PAM was proposed by analyzing the accumulation trends during the whole growing season. In comparison with the rice AGB estimation model based on VI and canopy height, PAM and SPAM presented in this study have stronger stability, better generalizability, and higher transferability, allowing for accurate and reliable estimates of rice AGB even when applied to data from different regions or locations. This study offers a reliable and useful tool to effectively predict the AGB of rice utilizing UAV digital and multispectral data, which has the potential to be a fast and quantitative approach for large-scale assessment of rice growth. In the future, these models are to be tested on samples of more rice cultivars and also to enhance the generality of the model through multi-year experiments.

## Data Availability

The data that support the findings of this study are available from the corresponding author upon reasonable request.
